# Impact of Magnesium on Oxytocin Receptor Function

**DOI:** 10.3390/pharmaceutics14051105

**Published:** 2022-05-21

**Authors:** Vimala N. Bharadwaj, Justin Meyerowitz, Bende Zou, Michael Klukinov, Ni Yan, Kaustubh Sharma, David J. Clark, Xinmin Xie, David C. Yeomans

**Affiliations:** 1 Department of Anesthesiology, Perioperative and Pain Medicine, School of Medicine, Stanford University, Palo Alto, CA 94304, USA; vimalanb@stanford.edu (V.N.B.); jmeyerow@stanford.edu (J.M.); klukinov@stanford.edu (M.K.); djclark@stanford.edu (D.J.C.); 2 AfaSci Inc., Burlingame, CA 94010, USA; bendezou@afasci.com (B.Z.); yanni@afasci.com (N.Y.); ksharma@afasci.com (K.S.); simonxie@afasci.com (X.X.); 3 Anesthesiology Service, Veterans Affairs Palo Alto Health Care System, Palo Alto, CA 94304, USA

**Keywords:** oxytocin, magnesium, pain, analgesia, headaches, agonist

## Abstract

**Simple Summary:**

What is already known: Mg^2+^ levels modulate the affinity of oxytocin receptors for oxytocin in vitro, low serum Mg^2+^ is correlated with migraine headache onset. What this study adds: Electrophysiologic and behavioral assays demonstrate that Mg^2+^ increases the efficacy of oxytocin; oxytocin efficacy is limited by Mg^2+^ availability. Clinical significance: Modulating Mg^2+^ levels may enhance oxytocin efficacy for pain, other uses, and endogenous processes.

**Abstract:**

Background and Purpose: The intranasal administration of oxytocin (OT) reduces migraine headaches through activation of the oxytocin receptor (OTR). Magnesium ion (Mg^2+)^ concentration is critical to the activation of the OTR, and a low serum Mg^2+^ concentration is predictive of a migraine headache. We, therefore, examined the functional impact of Mg^2+^ concentration on OT-OTR binding efficacy using two complimentary bioassays. Experimental Approach: Current clamp recordings of rat trigeminal ganglia (TG) neurons measured the impact of Mg^2+^ on an OT-induced reduction in excitability. In addition, we assessed the impact of Mg^2+^ on intranasal OT-induced craniofacial analgesia in rats. Key Results: While OT alone dose-dependently hyperpolarized TG neurons, decreasing their excitability, the addition of 1.75 mM Mg^2+^ significantly enhanced this effect. Similarly, while the intranasal application of OT produced dose-dependent craniofacial analgesia, Mg^2+^ significantly enhanced these effects. Conclusions and Implications: OT efficacy may be limited by low ambient Mg^2+^ levels. The addition of Mg^2+^ to OT formulations may improve its efficacy in reducing headache pain as well as for other OT-dependent processes.

## 1. Introduction

Oxytocin (OT) is a nine-residue, cyclized peptide which, through to the oxytocin receptor (OTR), acts as both a hormone, regulating diverse processes such as glucose metabolism, social bonding, lactation, and uterine contraction, as well as a neurotransmitter, modulating neural processes including pain [1,2,3,4]. Inadequate OTR activity is associated with postpartum hemorrhage, inadequate milk production, autism spectrum disorder (ASD), Prader–Willi syndrome, and migraine headaches [5,6,7,8]. Studies in both animal models and patients have demonstrated that intranasal administration of OT can relieve core symptoms in ASD, schizophrenia, migraine, and anxiety disorders [9,10,11]. Several factors have been found to alter OTR activity including OT levels, OTR expression, and OTR affinity for its ligand [8]. The last of these, OTR affinity, is closely modulated by the local concentration of the magnesium cation (Mg^2+^) which acts as an essential cofactor, dramatically increasing the affinity of OTR for OT [8,12,13]. Recently, Meyerowitz et al. [14] published the structure of the OT-OTR-Mg^2+^ coordination complex as identified using cryo-electron microscopy, demonstrating the critical role that Mg^2+^ plays in OT binding to OTR. Thus, alterations of serum or exogenously applied Mg^2+^ levels should play a critical role in OTR activity and consequently the physiological and psychological functions listed above.

Our previous work showed that OT decreases craniofacial pain in rodent models and headache severity and frequency in patients with migraine [10]. Not surprisingly, low serum Mg^2+^ levels strongly correlate with the frequency of headache attacks in patients with migraine [15,16,17]. However, the functional consequence of Mg^2+^ control of OT binding has not been adequately examined, particularly with regard to the function of OTR activity and pain. The purpose of this study was, therefore, to examine the impact of Mg^2+^ on OT-OTR binding efficacy by examining these effects in neuronal electrophysiology and pain behavior. The tools selected to address our question included both current clamp experiments on freshly dissociated rat trigeminal ganglia (TG) neurons to determine OT effects on neuronal excitability as well as the in vivo impact of the addition of Mg^2+^ to intranasally administered OT in a rat model of craniofacial pain.

## 2. Materials and Methods

### 2.1. Experimental Design

The objective of this study was to examine the impact of Mg^2+^ on OT-OTR binding efficacy by examining the effects on two key physiologic assays. We have previously demonstrated that OTR levels in trigeminal neurons are dependent, in part, on the presence of inflammatory cytokines [18]. Therefore, in this study, rats were pretreated with a complete Freund’s adjuvant (CFA) injection into the temporomandibular joint (TMJ) in order to produce a robust inflammation of trigeminally innervated tissue, inducing OTR upregulation approximately 24 h prior to harvesting and dissociating TG neurons. An electrophysiological recording was used to measure the change in membrane potential of TG neurons due to vehicle/OT treatment. In a separate group of animals, approximately 24 h after CFA injection, withdrawal latencies in response to noxious heat applied to the cheek were determined. Increased withdrawal latencies was indicative of analgesia/anti-nociception. Sample sizes were chosen on their basis to detect statistically significant results, with statistical analysis detailed throughout the study.

### 2.2. Animals

All animal care and experimental procedures complied with the laws of the United States and regulations of the Department of Agriculture and were approved by the Stanford University (Stanford, CA, USA) Institutional Animal Care and Use Committee, in accordance with the 2011 National Institute of Health Guide for the Care and Use of Laboratory Animals. Animal studies are reported in compliance with the ARRIVE guidelines (Kilkenny, Browne, Cuthill, Emerson, & Altman, 2010; McGrath & Lilley, 2015). Ethical permission (A3213-01 on 3 November 2020)

Rats (male, 250–330 g, Envigo, Indianapolis, IN, USA) were maintained two per cage in a controlled environment (temperature: 21.5 ± 4.5 °C/relative humidity: 35–55%) under a standard 12 h light/12 h dark lighting cycle (lights on at 7:00, no twilight). Cage changes occurred twice a week, using standard bedding. Food and water were provided ad libitum. Estrogen levels have been shown to drive OTR expression levels and so could drive substantial variability in response to OT treatment. Thus, we chose to use male animals for these studies to minimize this factor. We are currently pursuing separate experiments intended to explore this issue with regard to Mg^2+^ effects. Initial sample sizes were approximated by Power analysis, with animals assigned to groups randomly. Drug treatment experiments were conducted in a blinded fashion.

### 2.3. Sample Preparation

#### 2.3.1. Solutions for Electrophysiological Recording 

OT (Grindeks, Riga, Latvia) was dissolved in distilled water as 1 mM stock and diluted for external solutions. Different concentrations of OT (1.0, 3.0, 10, 30, 100, 300, or 1000 nM) were applied from the reservoir using gravity feeding through a 27G tip-blunted needle with an opening placed about 200 µm away from the recorded cell. The stream of solution covered the cell well when the switch was turned on. The cells were physically stable during perfusion.

#### 2.3.2. Solutions for Behavior Studies

The appropriate amount of test compound was accurately weighed out using a calibrated electrical balance and placed into microcentrifuge tubes. Solutions were prepared for an administration volume of 50 μL containing vehicle or one solution for one of five doses of OT (0.5, 1.0, 4.0, 8.0, or 32.0 μg) plus or minus 300 mM magnesium citrate. These doses and concentrations were selected based on the results of prior studies [10,19,20]. Ten groups of 10 rats each were randomly assigned to receive a vehicle or one of the five doses of OT or OT plus 300 mM Mg^2+^. Each solution was coded by the sponsor and the experimenter performed the experiment in a strictly blinded manner, including drug administration and data analysis.

### 2.4. Electrophysiology

#### 2.4.1. Induction of Inflammation 

Briefly, rats (male, 250–330 g, Envigo, n = 10) were placed in an anesthesia chamber and anesthetized with 2.5% isoflurane. Prior to TMJ injection, the rat’s mouths were propped open to palpate the target area. In this position, an oval-shaped groove located in the center of the cheek and above the mandible can be distinctly felt. With the syringe positioned at a 30-degree angle from the rat’s cheek, the tip of the needle was inserted just under the articular disc (approximately 1.5 mm in diameter and 1.0 mm deep). Thereafter, 50 µL of CFA (DIFCO; Sigma Aldrich, St. Louis, MO, USA) was injected (1 mL syringe with a 25G 5/8-inch needle) into the left TMJ to produce robust and prolonged orofacial inflammation. After CFA injection, rats were returned to home cages. Approximately 24 h later, rats were euthanized by decapitation after induction of deep anesthesia with isoflurane.

#### 2.4.2. Tissue Processing

The rat’s TG were carefully dissected from the surrounding connective tissues and minced into small pieces with an iris scissor. The TG were digested in 0.5 mL of mixed enzyme solution: (*w*/*v*, final concentration) 0.1% trypsin (Sigma, T9201), 0.1% collagenase Sigma, C1764), and 0.01% DNase (Sigma, D5025) diluted in Dulbecco’s Modified Eagle Medium/Nutrient Mixture F-12 (DMEM/F12) (Sigma, St. Louis, MO, USA). The tissue pieces were then incubated at 32 °C with a water bath for 55 min. Following digestion, tissue fragments were mechanically dissociated using a series of glass Pasteur pipettes with decreasing internal diameter. Dissociated cells were centrifuged at 180× *g* for 3 min, the supernatant was removed, and the cells were gently re-suspended in an external recording solution. Cells were then plated onto poly-l-lysine (Sigma, St. Louis, MO, USA) coated cover slips (Chemglass Life Sciences Vineland, NJ, USA.

#### 2.4.3. Current Clamp Recording

Whole-cell voltage-clamp recordings were performed using the MultiClamp 700B amplifier (Molecular Devices, San Jose, CA, USA) and analyzed offline with pCLAMP10.4 software (Molecular Devices, San Jose CA, USA). The external solution was composed of (in mM) NaCl (130), N-2-Hydroxyethylpiperazine-N’-2-Ethanesulfonic Acid (HEPES)-Na (10), KCl (5), CaCl_2_ (1), and Glucose (10), pH adjusted to 7.3–7.4 using HCl, with or without 1.75 mM MgCl_2_. The electrode internal solution was composed of (in mM) KF (120), HEPES (10), ethylene glycol-bis(β-aminoethyl ether)-N,N,N′,N′-tetraacetic acid (EGTA) (11), CaCl_2_ (1), MgCl_2_ (1), KCl (10), and KOH (11), pH adjusted to 7.3–7.4 using KOH. Patch-pipettes were fabricated from 1.5 mm outside diameter (OD) borosilicate capillary glass (Warner Instruments, Hamden, CT, USA) using a micropipette puller (Model P-87, Sutter Instrument, Novato, CA, USA). NaCl, HEPES-Na, KCl, CaCl2, Glucose, HCl, MgCl2, KF, HEPES, EGTA, KOH were purchased from Sigma (St. Louis, MO, USA). Glass pipettes filled this intracellular saline with a resistance of 3–5 MΩ. Whole-cell patch recordings had series resistances of <25 MΩ after whole-cell configuration and were periodically checked with the seal test voltage step (10 mV, 10 ms) to monitor series resistances throughout the recordings. Hyperpolarizing current pulses (about −0.3 nA, 500 ms) were delivered every 5 s throughout the experiment, unless otherwise specified, in order to monitor membrane input resistance and stabilize membrane potential in control external solution.

Measurement of change in membrane potential: After successful current clamp recording, the effect of the vehicle external solution application to cells on membrane potential was measured. After the membrane potential had stabilized for at least 10 s, a solution containing OT plus or minus 1.75 mM Mg^2+^ was applied for 2–5 min until the membrane potential stabilized further (on a new level) for at least 10 s. 

### 2.5. Behavioral Analgesia

#### 2.5.1. Withdrawal Latency

Rats (male, 250–330 g, Envigo, n = 10 per group) were used and treated with CFA injection into the TMJ as described above in order to produce a robust inflammation of trigeminally innervated tissue. Approximately 24 h after CFA injection, withdrawal latencies in response to noxious heat applied to the depilated (NAIR^®^ hair removal cream; Church & Dwight Co., Ewing, NJ, USA) and blackened (with India ink (Chartpak Inc., Leeds, MA, USA)) cheek were determined. Latency to withdrawal response was used as an indicator of nociceptive responsiveness. We have previously demonstrated, using single-fiber peripheral nerve recordings, that low intensity (slow ramp) skin heating evokes withdrawal responses mediated by the activation of C- (unmyelinated) nociceptive fibers; higher intensity skin heating (rapid ramp) selectively elicits responses mediated by A-delta (myelinated) thermonociceptors [21,22]. Briefly, to assess C fiber mediated responses, heat intensity was adjusted by altering the supply voltage (35–55 V) of the focused lamp until a withdrawal response was observed to occur with latency between 7.5 and 8.5 s. In order to reduce the potential for tissue damage, a cut-off latency of 15 s was implemented after which the stimulus was terminated. Rats not responding (within 10 s) to a supply voltage of 55 V during baseline testing were excluded from the study. For A-delta fiber testing, the heat intensity was adjusted by altering the supply voltage (60–85 V) of the focused lamp until withdrawal responses were observed to occur with a latency of between 2.5 and 3.5 s. The intensity applied to achieve such latencies was noted for each animal and used to assess withdrawal latencies prior to and following nasal administration of the test agent. To reduce the potential for tissue damage, a cut-off latency of 5 s was implemented during A-delta fiber testing. Rats not responding (within 3.5 s) to a supply voltage of 85 V were excluded from the study. A total of five rats were excluded from further testing by not reaching these criteria. Baseline withdrawal latencies were determined for each fiber type prior to nasal application of the test agent.

To deliver intranasal OT or vehicle the rats were anesthetized in a chamber using isoflurane (2%). They were then placed on a heating pad in a supine position as the anesthesia was continued with a nose cone. This horizontal position of the head was maintained throughout the procedure preventing drainage of the drug solution to the trachea and esophagus. The total volume of 50 μL solutions was administered by pipette in 6–7 μL drops in alternating naris every two min, over a total of 14 min. The drop was placed at the naris opening while occluding the opposite naris allowing the animal to snort the drop into the nasal cavity. The rats were allowed to wake up in a separate cage on a heating pad. Rats were then returned to their home cage. Withdrawal latencies in response to A-delta or C fiber cheek stimulation were then remeasured at 60 min following dosing. At the end of the testing session, rats were euthanized by CO_2_ inhalation.

#### 2.5.2. Efficacy Evaluation

Withdrawal latencies in response to thermal stimuli (noxious heat) were recorded as an index of thermal pain sensitivity. Increased withdrawal latencies were considered indicative of analgesia/anti-nociception.

### 2.6. Data and Statistical Analysis

All data are presented as means ±SD with significance set at *p* < 0.05. Statistical analysis was undertaken only for data sets where each group size was at least n = 5. All results were analyzed using the GraphPad Prism 9.0 software (GraphPad Software Inc., San Diego, CA, USA. RRID:SCR_008).

#### 2.6.1. Electrophysiology

Data were acquired using Clampfit—V10.4, Molecular Devices (San Jose, CA, USA) and data sheets were constructed in Excel (Microsoft) and GraphPad Prism Software. Two-way ANOVA were performed to compare the overall significance of the difference between OT and OT + Mg^2+^. Sidak’s multiple comparison test was used to determine differences at individual concentrations. Significance was set at *p* < 0.05.

#### 2.6.2. Withdrawal Latency

All data are expressed as mean withdrawal latency ±SE at 60 min post-dosing. Data generated during the testing of each fiber type (e.g., A-delta and C fibers) were analyzed, tabulated, and graphed separately. Statistical analyses were conducted using GraphPad Prism statistical software. All tests were conducted at the 0.05 level of statistical significance. Data generated were assessed using separate 2-way repeated-measures ANOVAs to determine if nasal OT produced dose-dependent significant increases in withdrawal latencies for A-delta or C fiber mediated responses and whether the addition of Mg^2+^ would significantly increase that response as determined by a significant difference between dose-response curves. Sidak’s multiple comparison tests were used for subsequent pairwise comparisons to pretreatment latencies.

## 3. Results

### 3.1. Electrophysiology Recording: Effect of Different Doses of OT with and without Mg^2+^ on Membrane Potential

**Figure 1:**
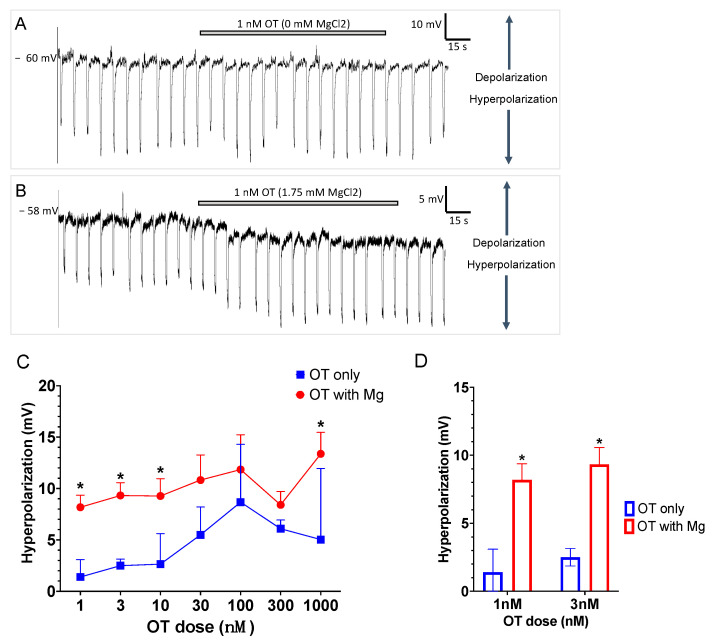
Effect of addition of Mg^2+^ to the OT-induced decrease in excitability of TG neurons from CFA-inflamed rats. (**A**,**B**) Examples of current clamp traces of TG neurons from CFA-inflamed rat; (**A**) no change in membrane potential when treated with 3 nM OT in a 0 mM MgCl_2_ buffer and (**B**) hyperpolarization observed when treated with 3 nM OT in a 1.75 mM MgCl_2_ buffer. (**C**,**D**) OT alone dose-dependently hyperpolarizes TG cell membranes, decreasing excitability; the addition of 1.75 mM Mg^2+^ significantly (* *p* < 0.05, ANOVA) potentiates the capacity of OT to hyperpolarize TG cell membranes (**C**). A specific example of this is observed in (**D**), where the addition of Mg^2+^ significantly (* *p* < 0.05) increased the induced membrane hyperpolarization of 1 nM or 3 nM OT from −1.4 ± 1.6 mV (n = 6) and −2.5 ± 0.6 mV (n = 6) for OT alone to −8.1 ± 1.2 mV (n = 7) and −9.3 ± 1.2 mV (n = 7) for OT plus Mg^2+^, respectively. Subsequent pairwise comparisons indicated significant (* *p* < 0.05) differences from OT alone for OT plus Mg^2+^ at 1, 3, 10, and 1000 nM OT concentrations. This enhanced hyperpolarization is emblematic of decreased excitability and thus, decreased capacity to carry pain signals to the central nervous system for pain perception. Error bars show ± S.D.

After a successful current clamp of TG neurons, the vehicle was applied as a control to the external solution. Both with or without 1.75 mM Mg^2+^, perfusion of cells with vehicle increased membrane potential insignificantly from −59.4 ± 1.9 mV to −60.6 ± 1.9 mV (*p* = 0.13, n = 9, student paired *t*-test). Figure 1A,B shows an example of a typical current clamp trace recording of a TG neuron from a CFA-inflamed rat. OT dose (3 nM) alone did not induce any change in the membrane potential (Figure 1A). However, the addition of 1.75 mM Mg^2+^ resulted in the hyperpolarization of membrane potential (Figure 1B). While OT alone dose-dependently induced membrane hyperpolarization, a consistently larger hyperpolarization of membrane potential was concentration-dependently observed with the addition of 1.75 mM Mg^2+^ (Figure 1C). Two-way ANOVA analysis showed the effect of OT on hyperpolarization was significantly different between the OT and OT + Mg^2+^ groups overall (2-way ANOVA, (*p* < 0.05)). A post-hoc multiple comparison (Sidak’s) test showed significant difference between OT and OT + Mg^2+^ groups at 1, 3, 10 (*p* < 0.05), and 1000 nM OT concentrations (*p* < 0.05).

In fact, while 1 or 3 nM OT had a minimal membrane potential effect (−1.4 ± 1.6 mV (n = 6) and −2.5 ± 0.6 mV (n = 6) hyperpolarization for 1 and 3 nM OT, respectively), the addition of 1.75 mM MgCl_2_ to the external solution induced a strong hyperpolarization, −8.1 ± 1.2 mV (n = 7 for 1 nM OT) and −9.3 ± 1.2 mV (n = 7 for 3 nM OT), respectively (Figure 1D). Two-way ANOVA analysis showed the overall effect of OT on hyperpolarization were significantly different between the OT and OT + Mg^2+^ groups (2-way ANOVA, (*p* < 0.05)). A post-hoc multiple comparison (Sidak’s) test showed a significant difference between OT and OT + Mg^2+^ groups at 1 (*p* < 0.05) and 3 nM OT concentrations (*p* < 0.05).

### 3.2. Behavioral Analgesia: Effect of Different Doses of OT with and without Mg^2+^ on Withdrawal Latency

**Figure 2:**
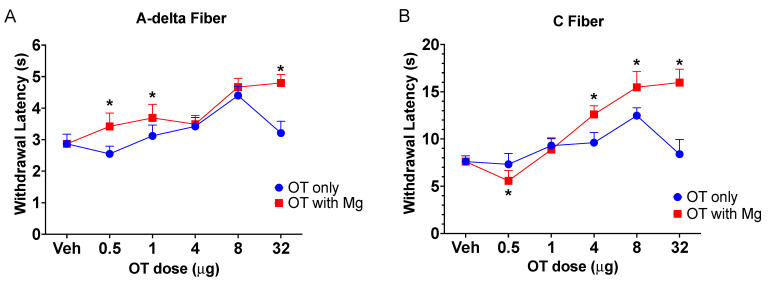
Effect of the addition of Mg^2+^ on intranasal OT-induced craniofacial analgesia. For both A-delta (**A**) and C fiber (**B**) mediated withdrawal responses to noxious heat stimulation of the cheek of pre-inflamed rats, intranasally applied OT produced a dose-dependent analgesic effect as evidenced by significant (*p* < 0.05, ANOVA) increases in withdrawal latency at the 60 min time point after administration, n = 10. However, the addition of 300 mM Mg^2+^ to the treatment significantly increased OT analgesia for both stimulus types (*p* < 0.05). Subsequent pairwise comparisons indicated significant (* = *p* < 0.05) differences from OT alone for OT plus Mg^2+^ for A-delta testing at OT doses of 0.5, 1.0, and 32 μg (*p* < 0.05); C-fiber responses were significantly different (* = *p* < 0.05) at 0.5, 4.0, 8.0, and 32 μg OT. Interestingly, while the efficacy of the highest OT dose (32 μg) demonstrated a decrease in efficacy when compared to lower doses, this dose-response inversion was prevented by the addition of Mg^2+^.

Baseline testing revealed stable head withdrawal response latencies for A-delta (2.6–3.2 s) and C-fiber (7.5–8.1 s) radiant heat stimulation of the cheek (Figure 2A,B). The intranasal application of OT produced a dose-dependent increase in withdrawal latency up to the highest dose (32 μg), where efficacy was seen to dramatically decrease for both A-delta and C fiber mediated pain responses. Application of the same doses of OT in the presence of Mg^2+^ however, produced a significant (*p* < 0.0001, 2-way ANOVA) increase in withdrawal latency compared to efficacy in the absence of Mg^2+^ for both A-delta (Figure 2A) and C-fiber (Figure 2B) testing. Follow-on analysis revealed significant differences (*p* < 0.05) between OT and OT + Mg^2+^ for A-delta testing at OT doses of 0.5, 1.0, and 32 μg OT; C-fiber responses were significantly different at 0.5, 4.0, 8.0, and 32 μg OT.

## 4. Discussion and Conclusions

The requirement of Mg^2+^ for OT’s high affinity for its receptor has long been known [13]. More recently, the precise architecture of this Mg^2+^ coordination site has been elucidated in the high-resolution cryo-electron microscopy structure of OT bound to its receptor, revealing that OT and OTR together form an octahedral Mg^2+^ coordination site between the receptor and ligand [14]. However, the functional consequence of Mg^2+^ control of OT binding has not been adequately examined, particularly with regard to how Mg^2+^ levels could affect the impact of OT-OTR binding on pain as well as other phenomena. The results of the second messenger study described in [14] demonstrate that Mg^2+^ is an essential cofactor for full OT-OTR agonism and that Mg^2+^ concentration-dependently increases G_q_ and G_11_ activation in OTR-transfected HEK cells. Interestingly, this study also demonstrated that OT-OTR binding did not reach maximal efficacy at physiologically relevant Mg^2+^ concentrations [14]. Consistent with these findings, while OT alone dose-dependently hyperpolarized TG neurons, decreasing their excitability, the addition of a supraphysiological (1.75 mM) concentration of Mg^2+^ significantly enhanced this effect, implying a supportive impact of Mg^2+^ on OT craniofacial analgesia. In a demonstration of this support, while intranasal application of OT produced dose-dependent craniofacial analgesia, the addition of 300 mM Mg^2+^ to the administered OT significantly enhanced this analgesia.

The trigeminal nerve provides pain signaling from the head to the central nervous system for the perception of craniofacial pain. Thus, decreases in the excitability of these neurons should produce decreases in craniofacial pain sensitivity. The results of the current study are consistent with our previous finding [23] that OT decreases the excitability of TG neurons in vitro as evidenced by a robust increased (hyperpolarized) cell membrane potential. This work also suggested that this decrease in neuronal excitability is likely mediated, at least in part, by an increase in voltage-gated K^+^ channel (Kv) current density [23]. As with the second messenger findings, the addition of 1.75 mM Mg^2+^ to the applied OT in the same concentration range used in the second messenger study produced a significant increase in the degree of hyperpolarization of the membrane. In the absence of Mg^2+^, the maximal efficacy of OT is not reached, indicating the necessity of Mg^2+^ for the full agonism of OT. Interestingly, while the efficacy of the highest OT dose (32 μg) demonstrated a decrease in efficacy when compared to lower doses, this inversion was prevented by the addition of Mg^2+^. These findings indicate that the addition of Mg^2+^ produces a more robust decrease in cell excitability, consistent with a stronger analgesic effect than that observed with OT alone.

Using autoradiography and tissue scintillation counts, we have previously demonstrated that radiolabeled OT, when applied nasally, concentrates in the trigeminal nerve and ganglia [20,24]; an approximately 10–20 times higher concentration of radiolabeled OT was detected in the trigeminal system compared to other tissue regions [20,24]. We have also shown that intranasal OT inhibits the transmission of pain messages to the central nervous system [10], inducing analgesia in rodent craniofacial pain models [10,19] and relief from headaches in patients with migraine [10]. The current study demonstrates that, as with the in vitro assays, the addition of Mg^2+^ to OT significantly enhances these analgesic effects. Previously, intravenous Mg^2+^ has been shown to abort continuous migraines and, when given as an oral supplement, reduce their frequency [25]. We have hypothesized that these effects might be mediated, in part, by a Mg^2+^ induced increase in the affinity of OTR for endogenous OT, thereby decreasing the excitability of trigeminal nociceptive neurons. Similarly, we have hypothesized that the decrease in serum Mg^2+^ during pre-menstruation and menstruation might help explain the phenomena of menstrual migraine [8].

In addition to the menstrual cycle effects on Mg^2+^ and migraine, serum estrogen levels, which vary over the menstrual cycle, have been shown to drive OTR expression levels [26,27] and have been hypothesized to underly, in part, the pathogenesis of menstrual migraine [8]. The variability of serum estrogen in females could also drive substantial variability in response to intranasal OT treatment. Thus, we chose to use male animals in these studies to minimize this factor. Because of the variability of serum Mg^2+^ and estrogen, it is likely that the effects of OT in females may vary significantly from those in males [8]. We are currently pursuing separate experiments intended to explore this issue with regard to the impact of Mg^2+^ on OT analgesia in females across the menstrual cycle.

The lowest OT dose (0.5 μg), supplemented with Mg^2+^ produced shorter withdrawal latencies compared to the OT group without Mg^2+^. One explanation for this observation is based on our preliminary electrophysiological studies that show 1.75 mM Mg^2+^, in the absence of OT, is in fact, depolarizing in some cells. Thus, with a very low concentration of OT, it is likely that this depolarizing effect overwhelms any minimal hyperpolarizing effect of the OT. Interestingly, while the efficacy of the highest OT dose (32 μg) demonstrated a decrease in efficacy when compared to lower doses, this inversion was prevented by the addition of Mg^2+^. In a separate ongoing study (and so not directly comparable), we have similarly found that 128 μg demonstrates a significant drop in efficacy compared to lower doses that were preventable by the addition of Mg^2+^. Inverted U dose responses have been widely reported for various systems, including social recognition [28], opioid-induced respiratory depression [29], and, very recently, in an autism spectrum clinical trial [30]. Although it is not unknown for peptide neurotransmitters to have an inverted U dose-response, the specific reason for a decrease in withdrawal latency at a higher OT dose is still unclear. One explanation could be off-target effects where OT, at high enough concentrations, begins acting on a different receptor. For example, OT has a very high affinity for the V1a receptor and so it is possible that the effects of OT on this or other receptors might counteract those on the OT receptor. This decrease in efficacy at higher doses of OT may be instrumental in the difficulty in demonstrating clear efficacy of intranasal OT in many clinical studies, where a moderate effect is seen with low doses, but higher doses do not show an improvement. The addition of Mg^2+^ to an OT formulation should allow the use of higher doses, overcoming this barrier for a number of indications. The second messenger study by Meyerowitz et al. [14] showed that the inversion of OT dose-response was not observed, which is likely due to the simplified milieu of the transfected cells versus that of whole neurons or in vivo.

Taken together, the results of these two sets of experiments suggest that Mg^2+^ is required for the full agonism of OT and that, for pain and in many other therapeutic or disease settings, the efficacy of OT may be limited by the availability of Mg^2+^. Thus, the addition of Mg^2+^ to OT formulations or the development of novel OT analogs based on recently elucidated OTR structural biology [14] that obviate the need for Mg^2+^ may enable enhanced OTR efficacy.

## Data Availability

The data that support the findings of this study are available from the corresponding author upon request.
